# Supplementary material for: Myosin-II mediated traction forces evoke localized Piezo1-dependent Ca2+ flickers
Source: Commun Biol. 2019 Aug 7;2:298. doi: 10.1038/s42003-019-0514-3 (PMC6685976; doi:10.1038/s42003-019-0514-3)
Supplement: Supplementary file 1 — Supplementary Information [file 42003_2019_514_MOESM1_ESM.pdf]

Supplemental Figures

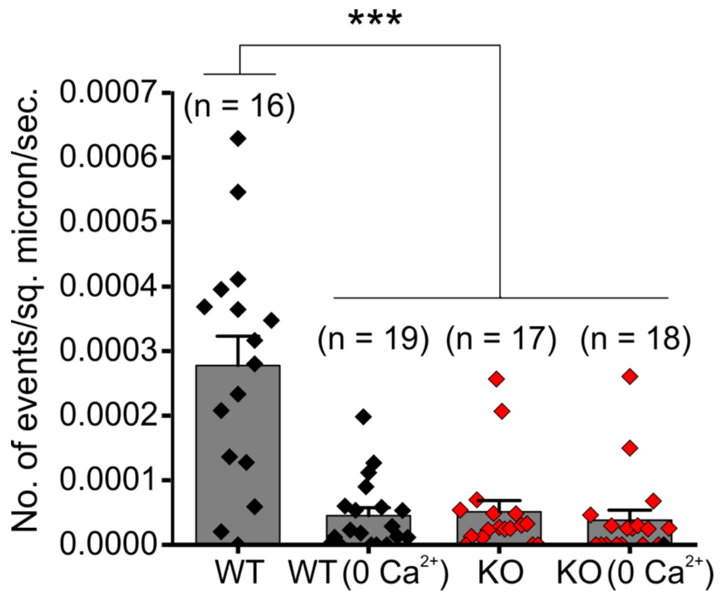

Supplementary Figure 1. Residual Ca^{2+} flickers in Piezo1-knockout HFFs are mediated by intracellular Ca^{2+} release. Related to Fig. 1. Ca^{2+} flickers in WT HFF cells are reduced in Ca^{2+}-free extracellular solution (denoted as "0 Ca^{2+}"). Piezo1-knockout HFF cells in standard 3 mM extracellular Ca^{2+} exhibit Ca^{2+} flickers to a similar extent as WT (0 Ca^{2+}) cells, and these residual flickers are not eliminated in Ca^{2+}-free extracellular solution. Taken together, these observations suggest that residual Ca^{2+} flickers in Piezo1-knockout HFFs occur due to intracellular Ca^{2+} release rather than plasma membrane ion channels. *** $p < 0.001$ by Kolmogorov-Smirnov test.

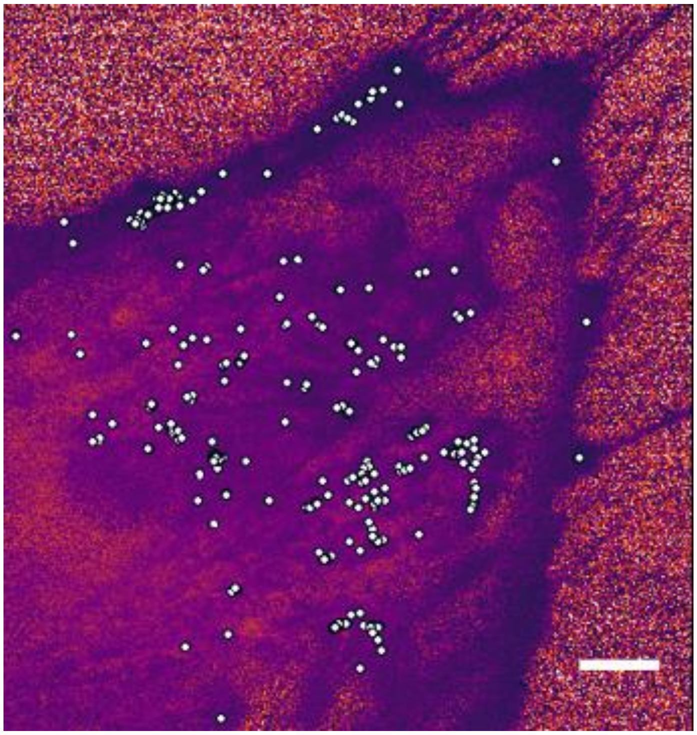

Supplementary Figure 2. Piezo1 flicker localizations extracted from Supplementary Movie 1. Related to Fig 2. Piezo1 Ca^{2+} flickers from HFF Supplementary Movie 1 were analyzed as described in Fig. 2 and Methods. Scale bar, 20 µm.

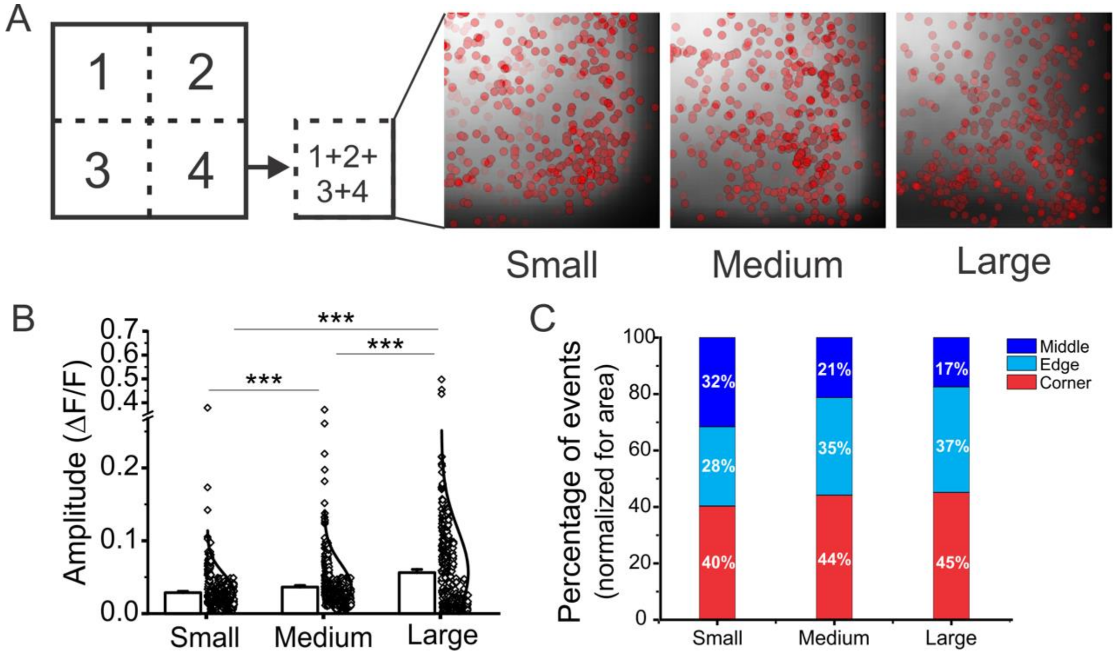

Supplementary Figure 3. Enrichment of Piezo1 Ca^{2+} flickers in Corners and Edges in square hNSPCs. Related to Fig. 3. A. Each panel shows a quadrant of an Cal520-loaded HFF, with an overlay of Piezo1 flicker localizations (red dots); since the square is a symmetric shape, flicker localizations from all four quadrants of cells are displayed on a single quadrant. Total numbers of events represented in panels C, D, E are: Small, 453 events from 7 cells; Medium, 476 events from 7 cells; Large, 426 events from 4 cells. The panels are shown scaled to the same size for ease of comparison. **B.** Bar and Data plot of flicker amplitudes (ΔF/F) for events from cells adhering to Small, Medium, and Large islands. Each dot represents an individual Piezo1 flicker event and bars denote the mean of all events for the specified cell size. Piezo1 Ca^{2+} flickers show greater amplitudes in cells seeded on large islands. *** denotes $p < 0.001$ by Kolmogorov-Smirnov test. **C.** Distribution of events in Corners, Edges and Middle regions for cells seeded on Small, Medium and Large squares. Difference of distribution patterns in hNPSCs and HFFs (see Fig. 3) suggests that the Piezo1 channel is tuned to different operating ranges in the two cell types. Chi-square test results: for Small cells □□ (2, N = 453) = 11.98, $p < 0.01$; for Medium cells □□ (2, N = 476) = 27.979, $p < 0.0001$; Large cells, □□ (2, N = 426) = 38.113, $p < .0001$.

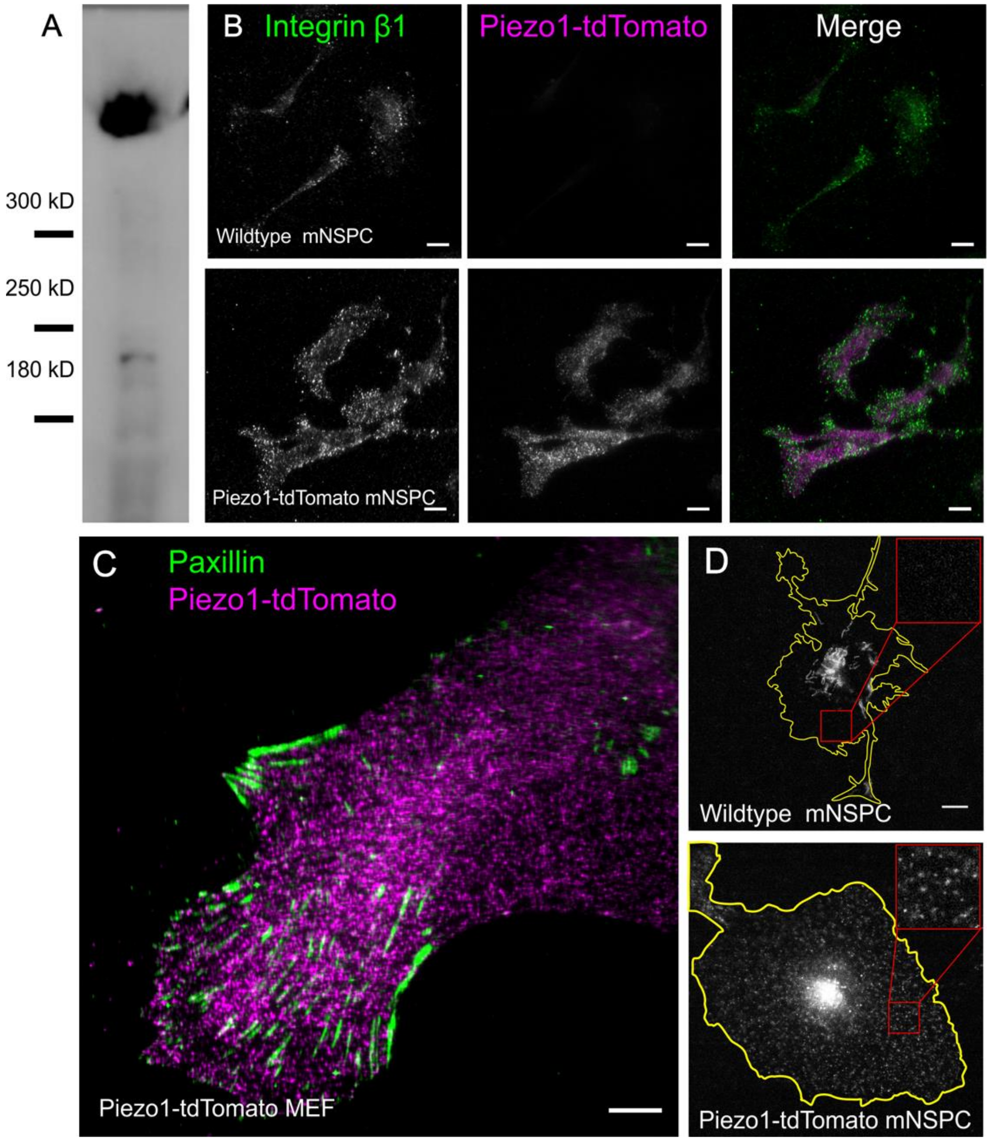

Supplementary Figure 4. Piezo1 localization is not restricted to integrin-rich focal adhesions. Related to Fig. 5. A. Western blot of lysate from Piezo1-tdTomato mNSPCs probed by an anti-RFP antibody shows a band of the molecular weight expected for a Piezo1 and tdTomato fusion protein (340 kD). **B.** Representative TIRFM images of mNSPCs immuno-labeled with antibodies against Integrin β1 (green) and tdTomato (red). The top row shows images of mNSPCs harvested from wildtype mice**,** and the bottom row shows images of mNSCPs harvested from Piezo1-tdTomato reporter mice. Scale bar = 10 µm. **C.** Representative TIRFM images of MEFs immuno-labeled with antibodies against focal adhesion protein paxillin (green) and tdTomato (red). Scale bar = 10 µm. **D.** Representative TIRFM images of tdTomato fluorescence of live mNSPCs from wildtype mice (top) and from Piezo1-tdTomato reporter mice. Inset shows magnification of a region of interest. While both cell types show autofluorescence (most prominent in the nuclear region), only Piezo1-tdTomato cells show small distributed puncta of tdTomato fluorescence. Scale bar = 10 µm.

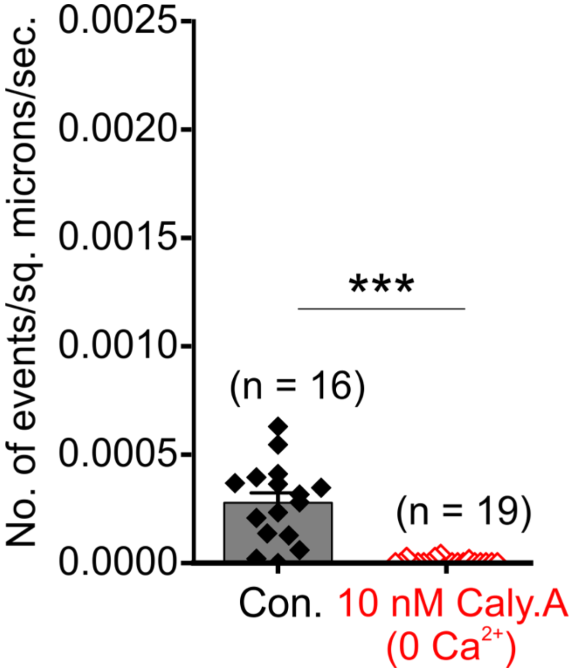

Supplementary Figure 5. Increase in Ca^{2+} flickers with Calyculin A requires external Ca^{2+}. Related to Fig 6. Flicker frequency in Control imaging solution and 1-5 minutes after replacing the control bath solution with 10 nM Calyculin A in Ca^{2+}-free imaging solution (i.e. standard imaging solution containing 2 mM EGTA and no $CaCl_2$). Bars denote Mean ± sem and each point represents flicker frequency in an individual video. Data are from three experiments. *** denotes $p < 0.001$ by Kolmogorov-Smirnov test.
