# Supplementary material for: Myosin-II mediated traction forces evoke localized Piezo1-dependent Ca2+ flickers
Source: Commun Biol. 2019 Aug 7;2:298. doi: 10.1038/s42003-019-0514-3 (PMC6685976; doi:10.1038/s42003-019-0514-3)
Supplement: Supplementary file 2 — Description of Additional Supplementary Files [file 42003_2019_514_MOESM2_ESM.docx]

**Description of Additional Supplementary Items**

**Supplementary Data 1. Source data file**

**Supplementary Movie 1. Piezo1 Ca^2+^ flickers are reduced in Piezo1-knockout HFFs.** The movie shows an F/F_0_ ratio movie of Ca^2+^ flickers from WT and Piezo1-KO HFFs.

**Supplementary Movie 2. Piezo1 Ca^2+^ flickers are reduced in Piezo1-knockout MEFs.** The movie shows an F/F_0_ ratio movie of Ca^2+^ flickers from WT and Piezo1-KO MEFs.

**Supplementary Movie 3.** **Piezo1 Ca^2+^ flickers imaged from HFFs.** The movie shows the F/F_0_ ratio movie from WT HFFs that was used for localization of Piezo1 Ca^2+^ flickers in Supplementary Fig. 2.

**Supplementary Movie 4. Mobility of Piezo1-tdTomato puncta in mNSPCs imaged using TIRFM.** A spatially filtered movie showing mobility of Piezo1-tdTomato channels in an mNSPC (yellow outline; puncta visible outside the yellow cell line are from neighboring cells).
