# Supplementary material for: Myosin-II mediated traction forces evoke localized Piezo1-dependent Ca2+ flickers
Source: Commun Biol. 2019 Aug 7;2:298. doi: 10.1038/s42003-019-0514-3 (PMC6685976; doi:10.1038/s42003-019-0514-3)
Supplement: Supplementary file 8 — Reporting Summary [file 42003_2019_514_MOESM8_ESM.pdf]

nature research

Corresponding author(s): Medha M. Pathak

Last updated by author(s): Jun 17, 2019

Reporting Summary

Nature Research wishes to improve the reproducibility of the work that we publish. This form provides structure for consistency and transparency in reporting. For further information on Nature Research policies, see Authors & Referees and the Editorial Policy Checklist.

Statistics

For all statistical analyses, confirm that the following items are present in the figure legend, table legend, main text, or Methods section.

n/a	Confirmed	
☐	☒	The exact sample size (n) for each experimental group/condition, given as a discrete number and unit of measurement
☐	☒	A statement on whether measurements were taken from distinct samples or whether the same sample was measured repeatedly
☐	☒	The statistical test(s) used AND whether they are one- or two-sided<br>*Only common tests should be described solely by name; describe more complex techniques in the Methods section.*
☒	☐	A description of all covariates tested
☐	☒	A description of any assumptions or corrections, such as tests of normality and adjustment for multiple comparisons
☐	☒	A full description of the statistical parameters including central tendency (e.g. means) or other basic estimates (e.g. regression coefficient) AND variation (e.g. standard deviation) or associated estimates of uncertainty (e.g. confidence intervals)
☐	☒	For null hypothesis testing, the test statistic (e.g. F, t, r) with confidence intervals, effect sizes, degrees of freedom and P value noted<br>*Give P values as exact values whenever suitable.*
☒	☐	For Bayesian analysis, information on the choice of priors and Markov chain Monte Carlo settings
☒	☐	For hierarchical and complex designs, identification of the appropriate level for tests and full reporting of outcomes
☒	☐	Estimates of effect sizes (e.g. Cohen's d, Pearson's r), indicating how they were calculated

Our web collection on statistics for biologists contains articles on many of the points above.

Software and code

Policy information about availability of computer code

Data collection	No custom software was used for data acquisition.
Data analysis	Custom analysis software has been deposited on GitHub (https://github.com/flika-org/flika).

For manuscripts utilizing custom algorithms or software that are central to the research but not yet described in published literature, software must be made available to editors/reviewers. We strongly encourage code deposition in a community repository (e.g. GitHub). See the Nature Research guidelines for submitting code & software for further information.

Data

Policy information about availability of data

All manuscripts must include a data availability statement. This statement should provide the following information, where applicable:

- Accession codes, unique identifiers, or web links for publicly available datasets
- A list of figures that have associated raw data
- A description of any restrictions on data availability

Data points are included in this article (and its supplementary information files) along with detailed methods, descriptions and sample movie files where appropriate. Moreover, source data underlying all graphs presented in the main figures are available online as a Supplementary Data file. Raw datasets generated and analyzed for the current study are available from the corresponding author on reasonable request.

Field-specific reporting

Please select the one below that is the best fit for your research. If you are not sure, read the appropriate sections before making your selection.

☒ Life sciences ☐ Behavioural & social sciences ☐ Ecological, evolutionary & environmental sciences

For a reference copy of the document with all sections, see nature.com/documents/nr-reporting-summary-flat.pdf

Life sciences study design

All studies must disclose on these points even when the disclosure is negative.

Sample size	The experiments in this paper were done in vitro at the single-cell level. We collected data from hundreds of Piezo1 flicker events from several cells (N numbers for each are indicated in figure legends) to give us confidence in the results. Additional details about sample size are provided in figure legends and Methods sections.
Data exclusions	Not applicable
Replication	Replicate information has been included in figure legends and Methods sections.
Randomization	Not applicable
Blinding	Blinding was performed by analysis software where appropriate (e.g. Flika, distance calculations etc).

Reporting for specific materials, systems and methods

We require information from authors about some types of materials, experimental systems and methods used in many studies. Here, indicate whether each material, system or method listed is relevant to your study. If you are not sure if a list item applies to your research, read the appropriate section before selecting a response.

Materials & experimental systems

n/a	Involved in the study	
☐	☒	Antibodies
☐	☒	Eukaryotic cell lines
☒	☐	Palaeontology
☐	☒	Animals and other organisms
☒	☐	Human research participants
☒	☐	Clinical data

Methods

n/a	Involved in the study	
☒	☐	ChIP-seq
☒	☐	Flow cytometry
☒	☐	MRI-based neuroimaging

Antibodies

Antibodies used	Rabbit anti-RFP (RFP Antibody Pre-adsorbed; Rockland, Cat# 600-401-379), 1:200 (0.95 µg/ml) and mouse anti-paxillin (clone 5H11, Millipore Cat # 05-417), 1:1000, mouse anti-Integrin (IGTB1; clone 2B1, Fisher Scientific cat # MA10690), 1:100. Secondary antibodies used were Goat anti-rabbit Alexa Fluor 555 (Invitrogen Cat# A21428) and Donkey anti-mouse Alexa Fluor 488 (Invitrogen, Cat# A-21202), and Goat anti-Mouse Alexa Fluor 488 (Invitrogen, Cat# A11029) were used at 1:200 (0.01 mg/ml).
Validation	No new antibodies were generated. All antibodies are commericially available and have been previously reported in the literature. Control experiments have been provided as supplemental data in the manuscript where appropriate.

Eukaryotic cell lines

Policy information about cell lines

Cell line source(s)	SC27 hNSPCs were previously derived (See Pathak et al. PNAS 2014 for details). mNSPCs and MEFs were cultured from embryonic mice as described in the Methods section. Human foreskin Fibroblasts (HFF-1) were purchased from ATCC (ATCC® SCRC-1041™). See Methods section for additional details on cell culture of each cell type.
Authentication	Commercial lines were purchased from the provider to ensure authentic lines. For non-commercial cell lines, low passage numbers of validated stock was used. Stringent cell culture practices to avoid cross contamination of cells was used.
Mycoplasma contamination	Mycoplasma tests were carried out with the cells and were negative.
Commonly misidentified lines (See ICLAC register)	No lines from the ICLAC table of commonly misidentified lines were used.

Animals and other organisms

Policy information about studies involving animals; ARRIVE guidelines recommended for reporting animal research

Laboratory animals	The study utilized Piezo1 reporter mice (JAX stock 029214), Piezo1 knockout mice (JAX stock 026948) and wildtype C57BL6 mice.
Wild animals	No wild animals were used.
Field-collected samples	No field-collected samples were used.
Ethics oversight	All studies were approved by the Institutional Animal Care and Use Committee at UCI.

Note that full information on the approval of the study protocol must also be provided in the manuscript.
